# Rice cultivation supports growth and survival of a threatened semi‐aquatic reptile

**DOI:** 10.1002/eap.70139

**Published:** 2025-12-08

**Authors:** Jonathan P. Rose, Allison M. Nguyen, Anna C. Jordan, Daniel A. Macias, Elliot J. Schoenig, Giancarlo R. Napolitano, Richard Kim, Julia S. M. Ersan, Alexandria M. Fulton, Brian J. Halstead

**Affiliations:** ^1^ U.S. Geological Survey Western Ecological Research Center Santa Cruz California USA; ^2^ U.S. Geological Survey Western Ecological Research Center Dixon California USA; ^3^ Present address: Marine Science Institute University of California Santa Barbara California USA; ^4^ Present address: Fish Program Washington Department of Fish and Wildlife Olympia Washington USA

**Keywords:** agroecosystems, integrated modeling, reconciliation ecology, working landscapes

## Abstract

Integration of agroecosystems and other working landscapes with protected lands and waters is critical to the conservation of Earth's biodiversity. Rice agroecosystems support many species by providing aquatic habitat where natural wetlands have been altered or drained. In regions with long dry seasons, rice fields and associated irrigation canals provide essential habitat for wetland‐dependent species. We quantified the spatial scale and magnitude of the effect of rice growing on the growth and survival of the giant gartersnake (*Thamnophis gigas*), a threatened species that persists primarily in areas of rice agriculture in the Central Valley of California, USA. We used structural causal models to identify drought condition as a key confounder to adjust for when estimating the total effect of rice growing on demographic rates. We analyzed capture‐mark‐recapture data from 19 populations of giant gartersnakes with an integrated growth–survival model and used distance‐weighted covariates to account for the decline in influence of rice with increasing distance from our study sites. We found strong support for a positive effect of rice grown within 1.9 km of a canal on giant gartersnake growth. There was also support for a positive effect of rice on giant gartersnake survival, although the spatial scale extended out to 5 km or more. Our results demonstrate how active rice growing benefits giant gartersnakes inhabiting irrigation canals and demonstrate an approach for studying landscape effects on wildlife in agroecosystems.

## INTRODUCTION

Agroecosystems are working landscapes that could play an important role in meeting biodiversity conservation goals (Kremen & Merenlender, 2018). These working landscapes can complement protected areas by providing connectivity for species to move between conserved lands (Perfecto & Vandermeer, 2008). Agroecosystems are not simply biodiversity corridors, however; they can support viable populations in situ, including threatened and endangered species (Estrada et al., 2012). Protected areas should, in theory, be more insulated from human exploitation driven by market forces (but see MacKenzie & Hartter, 2013). In contrast, species inhabiting agroecosystems are susceptible to changes in farming practices driven by commodities markets (Donald, 2004; Lenzen et al., 2012), which can be influenced by climate change (Fuhrer, 2003; Gurgel et al., 2021).

In dry climates, agriculture can depend on irrigation to supplement precipitation. If water is scarce during droughts, competition can prevent farmers from obtaining the water needed to grow crops (Medellín‐Azuara et al., 2022). High water prices also incentivize farmers to sell their allotment of water if profits will be greater from selling water than selling crops (Arellano‐Gonzalez et al., 2021). Wetland ecosystems in Mediterranean climates support tremendous biodiversity (Perennou et al., 2020; Taylor et al., 2021) that could be at risk if water is diverted for human use. Droughts in Mediterranean climates can affect wetland‐dependent species by reducing available habitat and food (Rose & Todd, 2017) and increasing disease risk (Adams et al., 2017).

Rice agriculture sits at the intersection of concerns about water availability, human land use, and biodiversity, because flooded rice fields provide wetland habitat for many aquatic and semiaquatic species (Bambaradeniya et al., 2004; Elphick, 2000). Although rice fields can act as surrogate habitat in the absence of natural wetlands, decreased rice growing driven by market forces threatens ecological communities dependent on these artificial wetlands (Koshida & Katayama, 2018). Conversion of land for agriculture has eliminated >90% of native wetlands in the Sacramento Valley (the northern part of the Central Valley) of California (Frayer et al., 1989; Garone, 2011). Rice growing covers more acres than any other crop in the Sacramento Valley during average to wet years (United States Department of Agriculture (USDA) National Agricultural Statistics Service (NASS), 2024), mitigating the loss of wetlands. Many species use rice fields and canals as habitat, including water birds (Elphick et al., 2008), reptiles (Fulton et al., 2022; Halstead et al., 2019), amphibians (Lawler, 2001), and fish (Holmes et al., 2021). During an extreme drought from 2013 to 2015, the amount of open water in rice fields and wetlands of the Central Valley declined (Reiter et al., 2018), and rice growing is expected to decline in the future under multiple climate change scenarios (Wilson et al., 2022). How declines in rice growing affect wildlife populations dependent on these artificial wetlands is a pressing conservation question.

As a threatened semiaquatic species endemic to the Central Valley, giant gartersnakes (*Thamnophis gigas*) might be particularly vulnerable to changes in rice growing driven by water scarcity. Although the species historically inhabited marshes, sloughs, and other slow‐moving bodies of water (Rossman et al., 1996), most extant giant gartersnake populations inhabit irrigation canals associated with rice agriculture (Halstead et al., 2010; Hansen et al., 2017), because canals often contain water for a longer hydroperiod than fields. The landscape surrounding habitat can influence animals' demographic rates including fecundity and survival (Moraga et al., 2019; Wright et al., 2019). For example, although radio‐tracked adult giant gartersnakes spend little time in rice fields (Halstead et al., 2016), the survival of large adult (predominantly female) snakes was positively related to the amount of rice grown near an individual's home range (Halstead et al., 2019). It is unclear however, if the putative benefits of rice growing apply to all life stages of giant gartersnake, because the size of radio transmitters only allows long‐term tracking of the largest adults. Capture‐mark‐recapture (CMR) models that integrate growth and survival are effective for estimating demographic vital rates of all life stages except neonate gartersnakes (Rose, Halstead, et al., 2018; Rose, Wylie, et al., 2018). Body size is related to demographic rates in many reptiles, including snakes (Shine & Charnov, 1992). Body size can influence fecundity (Weatherhead et al., 1999), growth (Howard et al., 2024), and survival (Hyslop et al., 2012); size distributions within populations can also affect population growth (Rose et al., 2019). Integrating growth and survival models can reveal insights into the demography of animals that would not be possible with either data type alone (Rotger et al., 2023) by enabling Bayesian imputation of individual size when animals are not captured (Bonner et al., 2010).

In this study, we analyzed CMR data from giant gartersnake populations in rice irrigation canals. We sought to address whether the area of active rice growing surrounding irrigation canals influences demographic rates of giant gartersnakes inhabiting canals. We used causal inference methods (Pearl, 1995) to identify covariates necessary to estimate the effect of rice growing on snake growth and survival. We then estimated the magnitude and spatial scale of the effect of rice growing on snake demographic rates in an integrated growth‐survival CMR model by employing a distance‐weighted rice covariate (Chandler & Hepinstall‐Cymerman, 2016; Miguet et al., 2017), while accounting for potential confounding effects of drought. Our results have implications for the management of water and cropping in the rice agroecosystem of the Sacramento Valley, conservation of an endemic species, and broader lessons for studying how landscape characteristics affect animal demography.

## METHODS

**FIGURE 1:**
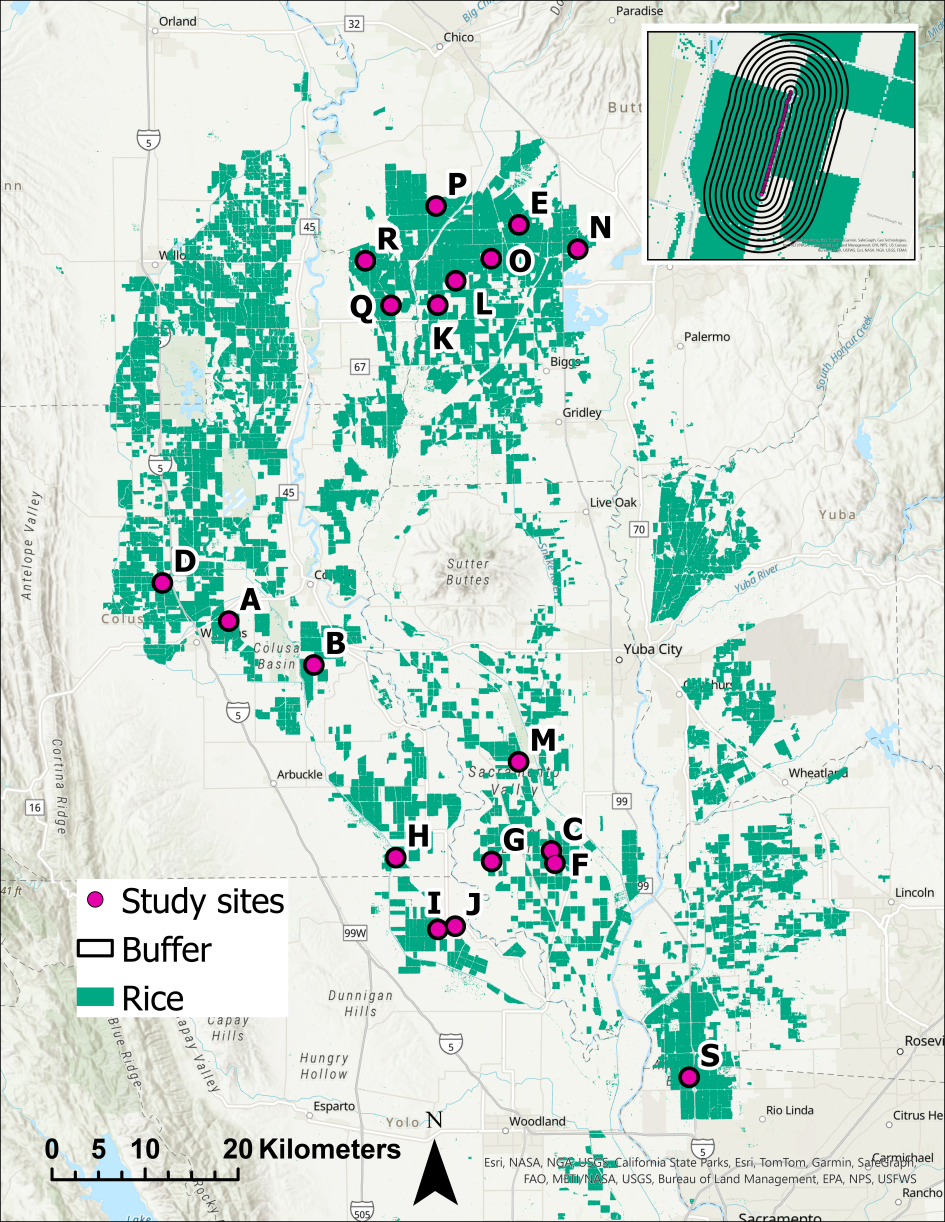
Study sites where giant gartersnakes (*Thamnophis gigas*) were sampled in the Sacramento Valley, CA, USA, from 2018 to 2023. Light green areas were classified as growing rice in the U.S. Department of Agriculture Cropland Data Layer (United States Department of Agriculture, 2024) for 2021. Buffers around traps in inset map range from 0.1 to 1 km in 0.1‐km increments for illustration; for analysis buffers ranging from 0.1 to 10 km were used.

We sampled giant gartersnake populations inhabiting rice irrigation canals at 19 sites in the Sacramento Valley, California, during 2018–2023 (Figure 1). At each site, we deployed traplines of 50 modified aquatic funnel traps (Casazza et al., 2000; Halstead et al., 2013) along the edge of the canal. Traps were spaced approximately 10–20 m apart, and traplines varied in length from 343 to 1414 m (mean = 761 m) depending on the length of canal available to sample. Eighteen sites were sampled each year during 2018–2023, and one site was only sampled during 2018–2022. The number of traplines deployed at a site varied among years depending on the availability of sufficient water to set traps and available personnel (Appendix S1: Table S1). We checked traps daily between 0900 and 1800 h. Traps were checked for an average of 27.6 days per site, per year (range = 12–76 days).

We marked all captured giant gartersnakes by branding a unique code on ventral scutes (Winne et al., 2006) and injected subcutaneous passive integrated transponder (PIT) tags (8 mm × 1.4 mm FDX‐B “Skinny,” Oregon RFID, Portland, OR, USA) in all snakes >30 g. We determined the sex of snakes by cloacal probing. We measured snake snout‐vent length (SVL) to the nearest millimeter by laying snakes along a meter stick. If snakes were captured and measured multiple times within a year, we only used the first measurement for analyzing year‐over‐year growth. This work was performed under U.S. Fish and Wildlife Service permits TE‐157216‐4 and TE‐157216‐5 and California Department of Fish and Wildlife Scientific Collecting Permit SC‐10779. All handling of snakes followed Animal Care and Use Committee protocol WERC‐2014‐01.

### Structural causal models

**FIGURE 2:**
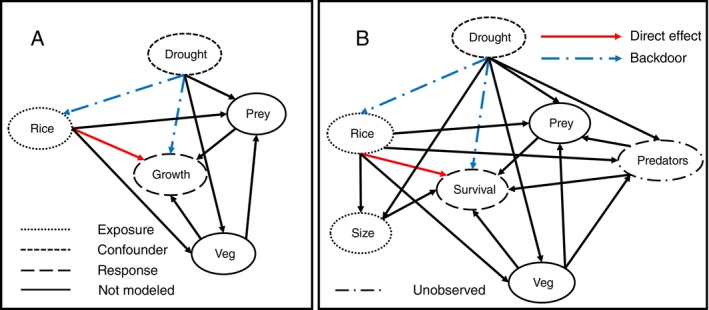
Directed Acyclic Graphs (DAGs) representing hypothesized causal models for covariate effects on giant gartersnake (*Thamnophis gigas*) growth (A) and survival (B). Rice is the exposure for which we desired to estimate the causal relationship with the response (growth or survival; red solid arrow), and drought is a confounder that is a common cause of both rice growing and the response and must be adjusted for in the model by closing the backdoor path (blue dashed arrow). Predators likely influence survival, but their abundance is unobserved; the effect of predators on survival can be ignored when estimating the total effect of rice on survival. Potential mediators for the effects of rice and drought on growth and survival are not included in the model because our goal was to estimate the total effect of rice on the response variables.

We created structural causal models (Pearl, 2009) with Directed Acyclic Graphs (DAGs) to represent our hypothesized relationships between giant gartersnake demographic rates and environmental conditions (Figure 2). We were interested in quantifying the relationships between the proportional area of rice grown around a site (exposure variable) and snake growth and survival (outcome variables). We tested for DAG data consistency (Arif & MacNeil, 2023) using the “dagitty” package (Textor et al., 2016) version 0.3‐4 in R version 4.4.0 (R Core Team, 2024) to evaluate if covariates assumed to be independent were not statistically associated. We then used dagitty to identify the sufficient set of variables to adjust to estimate the total effect of our exposure, rice growing, on the relevant outcome variable (growth or survival, depending on the model). We hypothesized that drought conditions could be a confounder that influences both the amount of rice grown and the growth (survival) of giant gartersnakes. Analysis of the DAGs identified an open backdoor path from rice to growth (survival) through the potential effects of drought on rice growing and snake growth (survival; Figure 2). Therefore, to estimate the effect of rice on growth (survival), drought must be adjusted for (i.e., included as a covariate) in the model.

As an index of drought conditions, we used the 6‐month Standardized Precipitation Evapotranspiration Index (SPEI06) based on the Pearson III distribution (Vicente‐Serrano et al., 2010). We downloaded gridded SPEI06 data from the National Centers for Environmental Information's (NCEI) “nClimGrid” monthly dataset (NOAA NCEI, 2024). We extracted monthly SPEI06 values for each of our study sites for 2018–2023 and included SPEI06 on August 1 of each year as our index of summer drought conditions. SPEI06 on August 1 integrates drought conditions over the preceding 6 months, from February through July. Negative values of SPEI indicate drier conditions than average and positive values indicate wetter conditions than average.

We used the “Cropland Data Layer” from the USDA NASS (2024) to calculate the area of rice grown in the landscape surrounding study sites for each year from 2018 to 2023. We created binary rasters for each year in which a value of one indicated rice was grown in that pixel (30 m resolution) and a value of zero indicated any other land use. For each site, we created a series of buffers surrounding the location of snake traps ranging from 0.1 to 10 km in 0.1‐km increments (Figure 1). We then extracted the number of pixels in which rice was grown within a ring of 0.1 km width corresponding to each buffer distance, calculated the area of rice grown, and divided by the total area of the buffer to calculate the proportion of the buffered ring (range 0–1) that had active rice grown for each site, in each year. We used the resulting matrix of 100 standardized rice values (raw proportions scaled to have mean = 0, SD = 1) for each site to calculate a distance‐weighted rice covariate effect on growth and survival following the methods of Chandler and Hepinstall‐Cymerman (2016) (Appendix S1). We then calculated two derived parameters to quantify the scale of effect for rice (Miguet et al., 2017): (1) The scale at which rice had the maximal effect on the response (scale_max_) and (2) the scale at which 90% of the cumulative effect of rice on the response is captured (scale_90_).

### Growth model

We modeled giant gartersnake growth using a modified von Bertalanffy growth function (Equation 1) for recapture data developed by Armstrong and Brooks (2013) and previously applied to San Francisco gartersnakes (*Thamnophis sirtalis tetrataenia*; Rose et al., 2022). In Equation (1), *L*_*i*,*t*−1_ is the measured SVL of snake *i* in year *t* − 1, EL_*i*,*t*_ is the expected length of snake *i* in year *t*, *a*_*i*_ is the asymptotic SVL for individual *i*, *k*_*i*,*t*_ is the growth rate of snake *i* in year *t*, and Δ*t* is the time interval between measurements. Because giant gartersnakes exhibit sexual size dimorphism (Rose, Halstead, et al., 2018), each individual snake's asymptotic SVL is determined by its sex (ε_*a*,sex_) and a sex‐specific random effect for site (λ_*a*,sex,*s*_; Equation 2). Although there can be individual variation in asymptotic length (King et al., 2016), we did not include an individual random effect on *a*_*i*_ in the model because preliminary model fitting indicated such a random effect was poorly identified, with the posterior distribution largely recapitulating the prior. For estimating the effect of rice growing on giant gartersnake growth, we limited our analysis to snakes that were captured and measured in consecutive years to isolate the effect of rice in a single year on growth. The log‐linear model for *k* (Equation 3) included a sex‐specific intercept (μ_*k*,sex_), the effect (β_rice_) of the distance‐weighted rice covariate around site *s* during year *t* (*R*_*w*(*s*,*t*)_) and the effect (β_drought_) of drought at site *s* in year *t* (SPEI_*s*,*t*_) on the growth coefficient *k* for year *t* to year *t* + 1. The model also included a sex‐specific random effect of site on *k* (α_*k*,sex,*s*_; Equation 4), an annual random effect on *k* (ζ_*k*,*t*_; Equation 5), and an individual random effect on *k* (ι_*k*,*i*_; Equation 6), where *N*(*x¯*, σ) is a normal distribution defined by mean (*x¯*) and SD (σ). *L*_*i*,*t*_ was modeled as arising from a normal distribution centered on the expected length (EL_*i*,*t*_) with an error term (σ_err_) that incorporates both measurement error and individual variation from expected length (Equation 7).

(1) $${\mathrm{EL}}_{i,t}=a_{i}-\left(a_{i}-L_{i,t-1}\right)\times e^{\left(\frac{k_{i,t}}{a_{i}}\times \Delta t\right)}$$

(2) $$a_{i}=\varepsilon_{a,\mathrm{sex}}+\lambda_{a,\mathrm{sex},s}$$

(3) $$\log\left(k_{i,t}\right)=\mu_{k,\mathrm{sex}}+\beta_{\text{rice}}\times R_{w\left(s,t\right)}+\beta_{\text{drought}}\times {\text{SPEI}}_{s,t}+\alpha_{k,\mathrm{sex},s}+\zeta_{k,t}+\iota_{k,i}$$

(4) $$\alpha_{k,\mathrm{sex},s}\sim N\left(0,\sigma_{s,k}\right)$$

(5) $$\zeta_{k,t}\sim N\left(0,\sigma_{t,k}\right)$$

(6) $$\iota_{k,i}\sim N\left(0,\sigma_{\mathrm{ind},k}\right)$$

(7) $$L_{i,t}\sim N\left({\mathrm{EL}}_{i,t},\sigma_{\mathrm{err}}\right)$$

We evaluated goodness‐of‐fit by comparing the discrepancy from expected values of snake SVL for observed data and a replicate dataset generated by the model based on RSS (Armstrong & Brooks, 2013).

### Integrated growth‐survival model

We fit an integrated growth–survival model by combining a robust‐design CMR model (Riecke et al., 2018) with the von Bertalanffy growth model described above (Equations (1), (2), (3), (4), (5), (6), (7)). The model conditioned on first capture of a snake and estimated sex‐specific parameters for daily recapture probability (*p*), availability for capture (γ), and apparent survival (ϕ) following the first capture. We included effects of rice (θ_rice_), and drought (θ_drought_) on ϕ, and a spline function to fit a nonparametric effect of snake SVL (*x*_*i*,*t*_) on ϕ, *f*_ϕ_(*x*_*i*,*t*_) (Equation 8). To account for variation among sites and years, we estimated a site‐ ($\rho_{\phi ,s}$; Equation 9) and year‐specific random effect on survival (η_ϕ,*s*,*t*_; Equation 10) to allow for deviations from the mean survival for each sex (μ_ϕ,sex_).

(8) $$\text{logit}\left(\phi_{i,t}\right)=\mu_{\phi ,\mathrm{sex}}+\theta_{\text{rice}}\times R_{w\left(s,t\right)}+\theta_{\text{drought}}\times {\text{SPEI}}_{s,t}+f_{\phi }\left(x_{i,t}\right)+\eta_{\phi ,s,t}$$

(9) $$\rho_{\phi ,s}\sim N\left(0,\sigma_{\phi ,s}\right)$$

(10) $$\eta_{\phi ,s,t}\sim \text{Normal}\left(\rho_{\phi ,s},\sigma_{\phi ,t}\right)$$

We did not include effects of snake size on recapture probability of snake *i* on day *j* in year *t* (*p*_*i*,*t*,*j*_) or availability of snake *i* in year *t* (γ_*i*,*t*_) in our final model because preliminary model fitting showed no evidence that either parameter was related to size (Appendix S1: Figure S1). Additionally, including size effects on *p* and γ did not alter the estimated relationship between size and ϕ, and simplifying linear models for these parameters greatly improved model run times. We fit sex‐specific intercepts for *p* (μ_*p*,sex_; Equation 11) and γ (μ_γ,sex_; Equation 12) and assessed evidence for an effect of sex by calculating the difference between estimates for males and females. We modeled random effects of site ($\upsilon_{p,s}$; Equation 13), year ($\delta_{p,s,t}$; Equation 14), and day‐within‐year (ξ_*s*,*t*,*j*_; Equation 15) on *p* and an annual random effect on γ ($\varsigma_{\gamma ,t}$; Equation 16).

(11) $$\text{logit}\left(p_{i,t,j}\right)=\mu_{p,\mathrm{sex}}+\xi_{s,t,j}$$

(12) $$\text{logit}\left(\gamma_{i,t}\right)=\mu_{\gamma ,\mathrm{sex}}+\varsigma_{\gamma ,t}$$

(13) $$\upsilon_{p,s}\sim N\left(0,\sigma_{p,s}\right)$$

(14) $$\delta_{p,s,t}\sim N\left(\upsilon_{p,s},\sigma_{p,t}\right)$$

(15) $$\xi_{s,t,j}\sim N\left(\delta_{p,s,t},\sigma_{s,t}\right)$$

(16) $$\varsigma_{\gamma ,t}\sim N\left(0,\sigma_{\gamma ,t}\right)$$

We evaluated goodness‐of‐fit for the robust‐design CMR model by calculating the expected number of recaptures for each snake in the years following its first capture and generating replicate recapture data from the model. We then calculated the Freeman‐Tukey discrepancy statistic comparing expected values to the observed and replicate recapture data, respectively (Rose et al., 2022).

We fit models using JAGS version 4.3.0 (Plummer, 2003) implemented in R version 4.4.0 (R Core Team, 2024) through the runjags R package version 2.2.2‐4 (Denwood, 2016). We adapted JAGS code for a distance‐weighted landscape covariate developed by Moll et al. (2020) for rice data surrounding our study sites (Appendix S1). We ran models on four independent chains for 250,000 iterations per chain, after discarding a burn‐in of 10,000 iterations. We thinned the resulting samples by a factor of 10, resulting in a final Markov Chain Monte Carlo (MCMC) of 100,000 samples for inference. We inspected trace plots to ensure chains were well mixed and looked for evidence of failure to converge. Both trace plots and the potential scale reduction factor (R‐hat; Brooks & Gelman, 1998) indicated no evidence of convergence failure (all parameters had R‐hat ≤1.01). Unless otherwise indicated, we report posterior means and 95% equal‐tailed credible intervals (95% CRI) to summarize parameter estimates. Data are archived on USGS ScienceBase (Rose et al., 2024), code necessary to reproduce growth and survival analyses are available on GitLab (Rose & Halstead, 2025) (refer to *Data availability statement*), and Appendix S1: Table S3 cross‐references parameter names in Equations ((1), (2), (3), (4), (5), (6), (7), (8), (9), (10), (11), (12), (13), (14), (15), (16))–((1), (2), (3), (4), (5), (6), (7), (8), (9), (10), (11), (12), (13), (14), (15), (16)) with names used in JAGS code.

### Model validation on simulated data

To validate the integrated CMR model's ability to accurately estimate *p*, ϕ, and γ, and the magnitude and scale of effect of rice on survival, we simulated data with randomly drawn, known parameter values, and analyzed those data with the model (Appendix S1). We evaluated accuracy by calculating the mean of the difference between the true value and estimated mean value for each simulation (bias) and determining the proportion of simulations in which the 95% CRI of the posterior distribution included the true value (coverage). We also simulated four different functional relationships between size and survival (size‐independent, linear, quadratic, and asymptotic) and tested if the spline function could accurately model the shape of each curve (Appendix S1).

## RESULTS

The area of rice growing varied among sites and years but on average increased with buffer distance from 0.1 km to 0.5–0.7 km and then decreased as buffer distance increased, with a gradual decline for buffers >4 km (Appendix S1: Figure S2). The average area of rice growing was fairly consistent during 2018–2021, but much lower in 2022, when several sites were surrounded by idle rice fields (Appendix S1: Figure S2).

Over 6 years of trapping at 19 sites, we made 3739 captures of 1785 individual snakes. Of these 1785 snakes, 987 were female, 781 were male, and 17 were of unknown sex. Most snakes (1501/1785) were only captured in 1 year. Of the recaptured snakes, 219 were captured in 2 years, 59 were captured in 3 years, 4 were captured in 4 years, and 2 were captured in 5 years, totaling 357 recaptures in years following the initial capture. Out of these 357 recaptures in a year subsequent to the first capture, 76.5% (*n* = 273) were made after 1 year, 19.6% (*n* = 70) were after 2 years, 2.0% (*n* = 7) were after 3 years, 1.7% (*n* = 6) were after 4 years, and one snake was first captured in 2018 and only recaptured in 2023. The most snakes were captured at sites A, J, L, O, and S (Table 1, Figure 1).

**TABLE 1:** Number of traplines deployed, number of snake captures, and number of individual giant gartersnakes (*Thamnophis gigas*) captured at each site from 2018 to 2023 in the Sacramento Valley, California, USA.

Sites	Years	Traplines	Captures	Individuals
A	2018–2023	3	244	129
B	2018–2023	2	130	76
C	2018–2023	3	114	54
D	2018–2023	1	175	123
E	2018–2023	3	93	63
F	2018–2023	3	26	13
G	2018–2023	1	13	8
H	2018–2023	1	91	47
I	2018–2023	3	57	30
J	2018–2023	4	427	177
K	2018–2023	1	131	73
L	2018–2023	2	1095	438
M	2018–2023	1	3	3
N	2018–2023	1	10	5
O	2018–2023	1	499	230
P	2018–2023	2	95	47
Q	2018–2022	2	116	64
R	2018–2023	1	15	7
S	2018–2023	5	405	198
Total	2018–2023	40	3739	1785

*Note*: Site code letters correspond to locations depicted in Figure 1. Traplines indicate the maximum number of traplines sampled at that site in a single year (see Appendix S1: Table S1 for variation in trapping effort by year).

**FIGURE 3:**
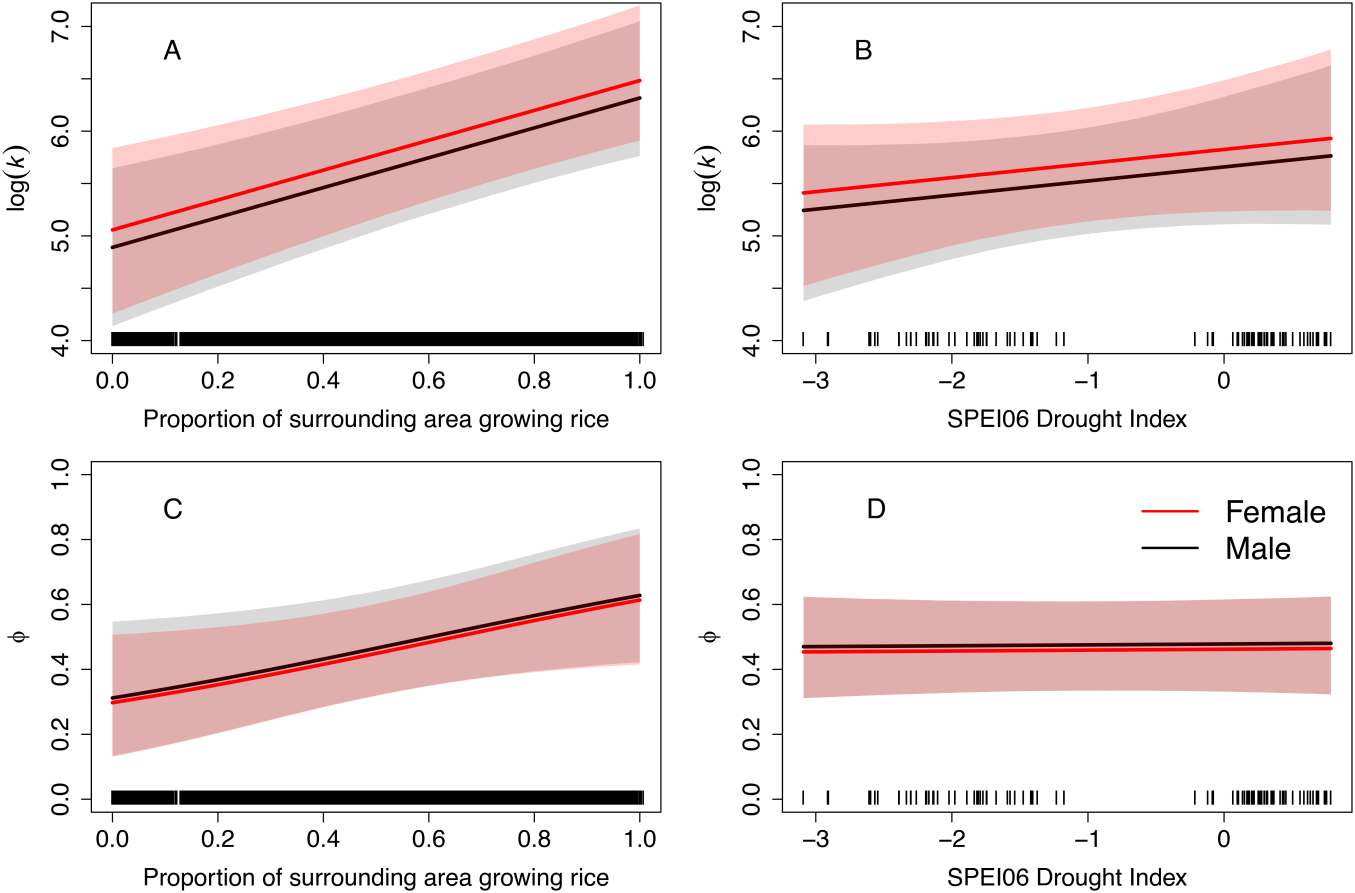
Relationship between giant gartersnake (*Thamnophis gigas*) growth (log(*k*), top row) and survival (ϕ, bottom row) and rice grown in the surrounding landscape (panels A and C) and drought conditions (Standardized Precipitation Evapotranspiration Index; SPEI06; panels B and D) in the Sacramento Valley, California, USA, 2018–2023. Lines represent posterior mean predicted relationships and shaded regions represent equal‐tailed 95% credible intervals. Tick marks above the *x*‐axis represent observed values for rice growing (A, C) and SPEI06 drought index (B, D).

**FIGURE 4:**
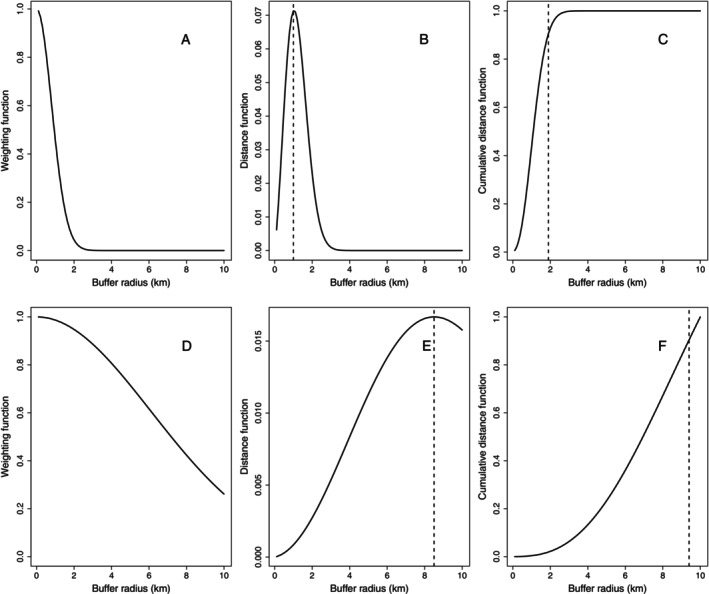
Scale of effect of proportion of rice grown in surrounding landscape on giant gartersnake (*Thamnophis gigas*) growth (*k* parameter in von Bertalanffy growth model; panels A–C) and survival (ϕ; panels D–F) in the Sacramento Valley, CA, USA, from 2018 to 2023. (A, D) Relative weighting of rice per unit area (*y*‐axis) as a function of buffer radius (*x*‐axis) for the effect of rice growing on *k*. (B, E) Distance function (*y*‐axis) versus buffer radius (*x*‐axis), with the dashed line representing scale_max_, the buffer radius at which rice growing is given maximum weight (1.0 km in B, 8.5 km in E). (C, F) The cumulative weight of rice as buffer radius increases, with a dashed line representing scale_90_, the spatial scale at which 90% of the cumulative weight is reached (1.9 km in C, 9.4 km in F).

From the 1785 captured snakes, 222 individuals had one or more recaptures in consecutive years, resulting in 267 growth intervals that could be used to fit the von Bertalanffy growth model. Females reached longer asymptotic lengths than males, but there was minimal difference in growth rates between the sexes (*p*[μ_*k*,male_ > μ_*k*,female_] = 0.79; Table 2). There was strong support for a positive effect of the area of rice grown on the growth rate of snakes that year (*p*[β_rice_ > 0] > 0.99; Figure 3). The scale parameter for the rice effect had a mean σ_rice,*k*_ = 0.8 km (95% CRI = 0.2–2.4 km). The buffer distance with the greatest weighting (scale_max_) was 1.0 km (0.3–3.2 km), and scale_90_ was 1.9 km (0.5–5.8 km), indicating a rapid decline in the influence of rice with distance (Figure 4). There was weak support for a negative effect of drought on growth (*p*[β_drought_ > 0] = 0.88), indicating slightly faster growth in wetter years (Table 2). The Bayesian *p*‐value for the growth model was 0.48, indicating no evidence of lack of fit.

**TABLE 2:** Parameter estimates from the von Bertalanffy growth model for giant gartersnakes (*Thamnophis gigas*) captured and measured in consecutive years in the Sacramento Valley, California, USA, 2018–2023.

Parameter	Description	Prior	Mean	SD	2.50%	97.50%	ESS
ε_*a*,fem_	Asymptotic SVL for females	N(900,200)	881.43	24.59	836.32	933.15	30,179
ε_*a*,male_	Asymptotic SVL for males	N(700,200)	645.32	16.93	614.27	681.30	28,901
μ_*k*,fem_	Log growth rate for females	N(0,10)	5.66	0.30	5.11	6.33	2809
μ_*k*,male_	Log growth rate for males	N(0,10)	5.83	0.31	5.23	6.49	3450
β_rice_	Effect of rice on *k*	N(0,10)	0.34	0.09	0.17	0.52	14,033
σ_rice,*k*_	Scale of rice landscape covariate	Unif(0.01,10)	0.80	0.70	0.22	2.36	8443
β_drought_	Effect of drought on *k*	N(0,10)	0.13	0.14	−0.09	0.47	2018
σ_ind,*k*_	SD of individual random effect on *k*	Exp(1)	0.35	0.06	0.23	0.48	12,543
σ_*t*,*k*_	SD of year random effect on *k*	Exp(1)	0.38	0.25	0.11	1.05	3827
σ_*s*,*k*_	SD of site random effect on *k*	Exp(1)	0.32	0.11	0.12	0.55	15,043
σ_*s*,*a*_	SD of site random effect on a	Exp(0.1)	37.58	11.66	14.35	61.57	14,485
σ_ind,*L*_	SD of individual variation in measured SVL	Exp(0.1)	26.23	2.05	22.48	30.52	19,580

*Note*: SD, standard deviation of the posterior distribution, 2.50% and 97.50% are the 2.5th and 97.5th percentiles of the posterior distribution, respectively. Parameter symbols correspond to those in Equations ((1), (2), (3), (4), (5), (6), (7))–((1), (2), (3), (4), (5), (6), (7)) in the main text. N(mean,SD) is a normal distribution with mean and SD, Unif(min,max) is a uniform distribution with minimum and maximum values, and Exp(rate) is an exponential distribution with a rate parameter.

Abbreviations: ESS, effective sample size for the Markov Chain Monte Carlo sample; SVL, snout‐vent length.

Apparent survival of giant gartersnakes was affected by rice growing (Table 3). There was support for a positive effect of rice growing on ϕ (*p*[θ_rice_ > 0] = 0.98), but no evidence for an effect of drought conditions on ϕ (*p*[θ_drought_ > 0] = 0.56; Figure 3). The spatial scale parameter for the effect of rice growing on ϕ (mean σ_rice,ϕ_ = 6.10, 1.03–9.82) was larger and more uncertain than the scale of effect for growth (Figure 4), with broad support for values ranging from 4 to 10 km, distances at which the change in the area of rice grown is more gradual (Appendix S1: Figure S2). Scale_max_ for the rice effect on survival was 8.5 km (1.4–10 km), and scale_90_ was 9.4 km (2.5–9.6 km), indicating a gradual decline in the influence of rice with distance (Figure 4).

**TABLE 3:** Parameters and posterior summaries from the robust‐design capture‐mark‐recapture model fit to data from giant gartersnakes (*Thamnophis gigas*) captured from 2018 to 2023 in the Sacramento Valley, California, USA.

Parameter	Description	Prior	Mean	SE	2.50%	97.50%	ESS
*p*_fem_	Daily recapture probability for females	Beta(1,1)	0.03	0.00	0.02	0.03	982
*p*_male_	Daily recapture probability for males	Beta(1,1)	0.02	0.00	0.02	0.03	989
σ_*p*,*t*_	SD of year random effect on *p*	Exp(1)	0.60	0.08	0.45	0.77	3070
σ_*p*,*s*_	SD of site random effect on *p*	Exp(1)	0.26	0.13	0.03	0.53	4631
θ_rice_	Effect of rice on ϕ	N(0,10)	0.33	0.18	0.02	0.72	2769
σ_rice,ϕ_	Scale of rice landscape covariate on ϕ	Unif(0.01,10)	6.10	2.47	1.03	9.82	23,465
θ_drought_	Effect of drought on ϕ	N(0,10)	0.01	0.08	−0.14	0.16	16,318
ϕ_fem_	Annual apparent survival for females	Beta(1,1)	0.48	0.08	0.33	0.65	5865
ϕ_male_	Annual apparent survival for males	Beta(1,1)	0.46	0.07	0.33	0.62	5748
σ_ϕ,*t*_	SD of year random effect on ϕ	Exp(1)	0.21	0.15	0.01	0.54	1477
σ_ϕ,*s*_	SD of site random effect on ϕ	Exp(1)	0.44	0.20	0.07	0.87	1983
γ_fem_	Availability for females	Beta(1,1)	0.55	0.08	0.40	0.71	16,398
γ_male_	Availability for males	Beta(1,1)	0.62	0.08	0.46	0.79	22,746
σ_γ,*t*_	SD of year random effect on γ	Exp(1)	0.73	0.35	0.11	1.51	2740

*Note*: Parameters include daily recapture probability (*p*), apparent survival (ϕ), and availability on site for capture (γ). Beta(alpha,beta) is a beta distribution with shape parameters alpha and beta, N(mean,SD) is a normal distribution with mean and SD, Unif(min,max) is a uniform distribution with minimum and maximum values, and Exp(rate) is an exponential distribution with a rate parameter.

Abbreviation: ESS, effective sample size for the Markov Chain Monte Carlo (MCMC) sample.

Snake size influenced survival in a nonlinear function. Adult snakes between 500‐ and 700‐mm SVL had the highest apparent survival, with decreasing survival for snakes <500 mm and snakes >700‐mm SVL (Appendix S1: Figure S3). Survival of neonate snakes <300 mm and large adult females >950‐mm SVL was highly uncertain because of small sample sizes. There was support for female snakes having a higher daily recapture probability than males (*p*[*p*_fem_ > *p*_male_] = 0.99), although the difference was small in terms of annual recapture probability. The cumulative annual recapture probability (p*, the probability of being recaptured at least once in a year following initial capture) for 21 days of sampling was 0.41 (0.35–0.48) for females and 0.38 (0.32–0.45) for males. There was little evidence of a difference between the sexes in annual apparent survival (*p*[ϕ_fem_ > ϕ_male_] = 0.62) or availability (*p*[γ_fem_ > γ_male_] = 0.27; Table 3). The Bayesian *p*‐value for the robust‐design CMR model was 0.18, indicating no evidence of a lack of fit to the data.

### Simulated data analysis

The robust‐design CMR model produced unbiased estimates of *p*, ϕ, and γ, and the posterior distributions for each parameter exhibited good coverage of the true values (Appendix S1: Table S2, Figure S4). For ϕ, 99 out of 100 simulations returned 95% CRIs that contained the true value (Appendix S1: Table S2). The spline function was adequate at estimating true relationships between snake size and survival for snakes between approximately 300‐ and 950‐mm SVL (Appendix S1: Figure S5). Uncertainty in size–survival relationships for the snakes <300‐mm SVL and >950‐mm SVL likely results from small samples of very small and very large snakes in the simulated datasets (Appendix S1: Figure S5).

The CMR model was able to accurately estimate β_rice_ for a range of plausible values, and 96 out of 100 simulations returned 95% CRIs containing the true value (Appendix S1: Table S2, Figure S6). Coverage was also high for σ_rice_ (98/100 simulations), but the precision of estimates of σ_rice_ depended on the value of this scale parameter. When σ_rice_ was <3 km, mean estimates were close to the true value and posterior distributions were precise, with narrow CRIs. For true values of σ_rice_ >3 km, the CRIs were very large, representing posterior distributions that had broad support for a range of possible scales (Appendix S1: Figure S6).

## DISCUSSION

Our results demonstrate the importance of estimating the scale of effect for landscape variables (rather than assuming the scale a priori), because the scale can depend on the response variable measured (Moraga et al., 2019). The differing scales of effect of rice on growth (1–2 km) and survival (≥4 km) were both larger than home range estimates (95% minimum convex polygons) from radio‐tracked giant gartersnakes inhabiting rice irrigation canals, which averaged 0.18 km^2^ (Nguyen et al., 2024). The larger scales of effect indicate that although a snake's movements during the active season might be restricted to within a few hundred meters of a canal, the broader landscape influences demographic rates of individuals in that canal. This has implications for managing rice agroecosystems, including whether keeping canals flooded during the active season is sufficient to sustain populations when rice fields are fallowed or rice is replaced by another crop. How the area of rice cultivation surrounding canals affects giant gartersnake growth and survival, and why the spatial scale of maximal effect differed between the two, remain open questions.

Despite rice providing surrogate wetlands in the summer, most adult giant gartersnakes spend little time in rice fields, instead inhabiting adjacent irrigation canals (Halstead et al., 2016, 2019; Nguyen et al., 2024). Rice growing could indirectly affect giant gartersnake growth and survival by increasing the abundance of prey. Rice provides wetlands that benefit fish and anuran communities (Holzer et al., 2017; Koshida & Katayama, 2018), and their invertebrate prey (Lupi et al., 2013). For example, a study of rice agroecosystems in Japan found irrigation canals with greater connectivity to rice fields supported higher fish diversity and abundance of flying insects (Katano et al., 2003). Increased prey abundance might explain the influence of rice growing within 1–2 km on snake growth, because the habitat adjacent to canals is the most likely source for prey subsidies moving into the canal. One potential explanation for the effect of rice on snake survival is that when rice fields are fallow, snakes experience increased mortality from predators, which can focus their foraging in relatively simple, linear canals (Halstead et al., 2019). Increased predation pressure in isolated canals could explain the influence of the wider landscape on survival. If the landscape surrounding canals is dry, predators might focus their foraging on canals where prey is concentrated. In contrast, if a canal is embedded in a landscape of extensive flooded rice, there could be less incentive for predators to focus on canals where aquatic prey might be concentrated. Further exploration would be needed to identify the variables that directly lead to faster growth and higher survival in snakes inhabiting landscapes with more rice. Regardless of the proximate causes of improved demographic rates, allocating water for growing rice and cultivating rice instead of other crops are decisions that can be influenced by state and federal water agencies, local water districts, and farmers (within parameters determined by broader precipitation and water use patterns). Increasing prey abundance within canals or decreasing predation pressure, without manipulating rice growing, could be challenging at a landscape scale.

The primary focus of inference in our CMR model was apparent survival (ϕ), which is the product of true survival and site fidelity (1—the probability of permanent emigration). The mean estimate of survival for female snakes (0.46) was lower than estimates based on radio‐tracked adult female giant gartersnakes (0.61; Halstead et al., 2012, 2019). Because we cannot disentangle permanent emigration from survival, the lower apparent survival of snakes in areas where less rice is grown could indicate snakes dispersing away from canals that are no longer adjacent to flooded rice. Permanent emigration from canals with minimal rice in the surrounding landscape might also explain the greater spatial scale of the rice effect on survival, if snakes are more likely to disperse from isolated canals without rice nearby. However, research into how rice growing affects giant gartersnake movement does not support permanent emigration as the cause of reduced apparent survival. A radiotelemetry study of giant gartersnakes found that adult females were more likely to move long distances (>100 m) and moved greater distances per day on average when more rice was actively growing within 100 m of their location (Reyes et al., 2017). Although we accounted for temporary emigration, the nonspatial CMR model used here cannot account for permanent emigration, and the apparent survival values reported here likely underestimate true survival. Spatially explicit capture–recapture models hold promise for estimating true survival while accounting for the movement of snakes on or off sampled areas (Gardner et al., 2010). These spatial models require repeated recaptures of individuals to estimate activity centers, which can be challenging for elusive animals like snakes (Steen, 2010).

Giant gartersnakes exploit irrigation canals associated with rice agriculture, but these habitats could be suboptimal compared to the wetlands the species historically inhabited (Halstead et al., 2012). Elucidating how canals and wetlands differ could provide guidance for improving management of rice canals and for the creation of wetland reserves for this threatened species. The size‐dependent survival curve for snakes inhabiting canals, with the highest survival for adult snakes of intermediate size, is similar to that from an earlier study of giant gartersnakes primarily inhabiting wetlands (Rose, Wylie, et al., 2018). However, survival in canals might be lower than in more natural wetlands (Halstead et al., 2012).

Conservation of aquatic species native to the Central Valley will involve wetlands (including canals) that are resilient to climate change and mimic historical floodplains (Power et al., 2024). The persistence of giant gartersnakes in the Sacramento Valley is attributable, in part, to the widespread irrigation of rice fields and canals that act as surrogate wetlands (Halstead et al., 2021). Although rice growing has aided the persistence of this threatened species, its reliance on agriculture also presents risks, if cultivation and land management practices change in the future (Halstead et al., 2019). The fate of other species dependent on aquatic habitat in the Central Valley is subject to land and water management (Central Valley Joint Venture, 2020). Many waterbird species depend on flooded rice fields for food during their fall migration through the Central Valley (Elphick, 2000), and the suitability of fields is affected by postharvest management (Miller et al., 2010). Inundated rice fields can provide high‐quality winter rearing habitat for juvenile Chinook salmon (*Oncorhynchus tshawytscha*), because the hydrologic conditions and high food abundance approximate historical floodplains (Holmes et al., 2021; Jeffres et al., 2020). Our results, combined with earlier work (Halstead et al., 2012, 2014), indicate that like other wetland‐dependent species in the Central Valley, the long‐term persistence of giant gartersnakes would likely benefit from a connected landscape of irrigated rice fields, canals, and natural and restored wetlands.

## CONFLICT OF INTEREST STATEMENT

The authors declare no conflicts of interest.

## Supporting information


Appendix S1.

## Data Availability

Data (Rose et al., 2024) are available in the USGS ScienceBase repository at https://doi.org/10.5066/P1JHAWVT. Code (Rose & Halstead, 2025) is available in the USGS GitLab repository at https://doi.org/10.5066/P145MXF4.
