# Supplementary material for: Rice cultivation supports growth and survival of a threatened semi‐aquatic reptile
Source: Ecol Appl. 2025 Dec 8;35(8):e70139. doi: 10.1002/eap.70139 (PMC12683694; doi:10.1002/eap.70139)
Supplement: Supplementary file 1 — Appendix S1. [file EAP-35-e70139-s001.pdf]

Appendix S1

Supplemental material for "Rice cultivation supports growth and survival of a threatened semi-aquatic reptile"

Jonathan P. Rose, Allison M. Nguyen, Anna C. Jordan, Daniel A. Macias, Elliot J. Schoenig, Giancarlo R. Napolitano, Richard Kim, Julia S.M. Ersan, Alexandria M. Fulton, and Brian J. Halstead

Ecological Applications

Acknowledgments

We thank the U.S. Bureau of Reclamation, The Natomas Basin Conservancy, and the U.S. Geological Survey's Ecosystems Mission Area for funding this work. We thank many landowners for providing access to their properties to sample for giant gartersnakes, and several water districts for coordinating access. This work would not have been possible without the diligent work of many biological technicians. Any use of trade, firm, or product names is for descriptive purposes only and does not imply endorsement by the U.S. Government.

Supplemental Methods

Distance-weighted rice covariate

We calculated a distance-weighted rice covariate effect on growth and survival following the methods of Chandler and Hepinstall-Cymerman (2016). In Eq. S1, $rice_{m(s)}$ is the proportion of the area within a buffer ring m at site s that grew rice, w_m is the weight given to buffer ring m, and $R_{w(s)}$ is the weighted rice covariate used in the model. The value of w_m is based on a Gaussian kernel where d is the radius of buffer m, A_m is the area encompassed within buffer ring m, and σ_{rice} is a scaling parameter that determines how the weight of the rice covariate declines with distance from the site (Eq. S2).

(S1) $$R_{w(s)} = \sum_m w_m \times rice_{m(s)}$$

(S2) $$w_m = \frac{exp\left(-\frac{d^2_{m(s)}}{2\sigma^2_{rice}}\right) \times A_m}{\sum_m exp\left(-\frac{d^2_{m(s)}}{2\sigma^2_{rice}}\right) \times A_m}$$

The value of σ_{rice} is estimated simultaneously with the magnitude of the effect of rice on the response variable in the hierarchical models for growth and survival described below. We then calculated two derived parameters to quantify the scale of effect for rice (Miguet et al.

2017): 1) The scale at which rice had the maximal effect on the response ($scale_{max}$), and 2) The scale at which 90% of the cumulative effect of rice on the response is captured ($scale_{90}$).

Data simulation

We simulated capture-mark-recapture (CMR) data using plausible parameter values similar to those estimated from our study populations. For the first simulation of CMR data with size-independent survival, true values of *p*, φ, and γ were randomly drawn from uniform distributions with a shared value for both sexes (Table S2). We then simulated data with fixed values for *p* (0.035), ϕ (0.45), and γ (0.65) and four potential relationships between snake size and survival: 1) Survival is size-independent, 2) A positive linear relationship, 3) A quadratic relationship with peak survival for intermediate sizes, and 4) A positive relationship that asymptotes at peak of 0.6 for large adults and a minimum of 0.2 for neonates. Data were simulated by adapting code developed by Riecke et al. (2018). All four size-survival relationships were used to generate 100 simulated CMR datasets similar in size to our giant gartersnake data (approximately 1,800 individuals sampled over six years, with 21 days of sampling per year). Snakes started the simulation with snout-vent lengths (SVLs) drawn from the observed distribution of SVL measurements in the field-collected CMR data. The integrated CMR model was fit to each simulated data set using a smaller MCMC sample (four chains, 2,500 sampling iterations following a burn-in of 1,000) to enable fitting 100 models in a tractable length of time. Finally, to evaluate the accuracy of model predictions of the magnitude and scale of effect for rice on ϕ we simulated 100 datasets in which true values of β_{rice} and σ_{rice} were drawn from uniform distributions (Table S2). Because estimating σ_{rice} requires a longer sampling period, we fit models on simulated rice data for a burn-in period of 10,000 iterations followed by 25,000 sampling iterations, which were thinned by a factor of 10. Code to simulate CMR data is available in Rose and Halstead (2025).

Description of R code and data files for reproducing analyses

Repository files

Available in Rose and Halstead (2025) at https://doi.org/10.5066/P145MXF4

* Run files in the following order:

1. `ms_functions.R` - Loads functions required to analyze data and run simulations.

2. `ms_load_data.R` - Loads and formats growth, capture-mark-recapture, rice, and drought data.

3. `ms_analysis.R` - Fits growth and capture-mark-recapture models to empirical capture data for giant gartersnakes.

4. `ms_simulations.R` - Simulates capture-mark-recapture data and fits models to validate parameter estimates.

5. `ms_figures.R` - Reproduces figures from the manuscript.

Data files

Available in Rose et al. (2024) at https://doi.org/10.5066/P1JHAWVT

Input data files include:

- `all_dates_array.R` - A `.R` file with an array of dates sampled (cell values) by site (second dimension) and year (third dimension).
- `all_site_years_trapped_df.R` - A `.R` file with the posterior distribution from a robust-design model to estimate the relationship between snake SVL and survival using non-linear splines. This file includes:

    - site_code - Letter representing the site depicted in Figure 1 of manuscript.

    - site_num - Numeric site indicator used to index data for models.

    - nyears.trapped - The number of years that giant gartersnakes were sampled at that site.

    - start.year - The first year in which a site was sampled.

    - last.year - The last year in which a site was sampled.

    - K.2018 - The number of days of sampling at each site in 2018.

    - K.2019 - The number of days of sampling at each site in 2019.

    - K.2020 - The number of days of sampling at each site in 2020.

    - K.2021 - The number of days of sampling at each site in 2021.

    - K.2022 - The number of days of sampling at each site in 2022.

    - K.2023 - The number of days of sampling at each site in 2023.

- `all_CH_2018-2023.R` -A`.R` file with the capture history for each snake. This file includes:

  - snake_ID - A numeric ID for each snake captured in the study.

  - site_code - A letter representing the site at which that snake was captured.

  - site_num - A numeric index for the site at which a snake was captured.

  - Columns for individual dates with binary indicators of captured (1) or not captured (0)

- `all_snakes_SVL.R`- A `.R` file with the sex and snout-vent lengths (SVL) in mm from captured snakes. This file includes:

  - snake_ID - A numeric ID for each snake captured in the study.

  - site_code - A letter representing the site at which that snake was captured.

- site_num - A numeric index for the site at which a snake was captured.

- sex - Indicates if a snake was female (F), male (M), or unknown (NA).

- male - Binary indicator of male sex (1), female (0), or unknown (NA).

- nSVL - The number of SVL measurements recorded for that snake.

- SVL.2018 - The SVL in mm for that snake in 2018, if captured and measured. NA if not captured and measured that year.

- SVL.2019 - The SVL in mm for that snake in 2019, if captured and measured. NA if not captured and measured that year.

- SVL.2020 - The SVL in mm for that snake in 2020, if captured and measured. NA if not captured and measured that year.

- SVL.2021 - The SVL in mm for that snake in 2021, if captured and measured. NA if not captured and measured that year.

- SVL.2022 - The SVL in mm for that snake in 2022, if captured and measured. NA if not captured and measured that year.

- SVL.2023 - The SVL in mm for that snake in 2023, if captured and measured. NA if not captured and measured that year.

* `ggs_growth_1year_increments_2018-2023.R` - A `.R` file with the number of captures and SVL measurements for snakes captured and measured in consecutive years. This file includes:

- snake_ID - A numeric ID for each snake captured in the study.

- site_code - A letter representing the site at which that snake was captured.

- site_num - A numeric index for the site at which a snake was captured.

- sex - Indicates if a snake was female (F), male (M), or unknown (NA).

- male - Binary indicator of male sex (1), female (0), or unknown (NA).

- ncaps - The number of captures and SVL measurements recorded for that snake.

- SVL1 - The SVL in mm for the first capture of that snake.

- SVL2 - The SVL in mm for the second capture of that snake.

- SVL3 - The SVL in mm for the third capture of that snake. Value is NA if the snake was not measured three times.

- SVL4 - The SVL in mm for the fourth capture of that snake. Value is NA if the snake was not measured four times.

- SVL5 - The SVL in mm for the fifth capture of that snake. Value is NA if the snake was not measured five times.

- year1 - The year (from 1-6) of the first measurement of SVL.

- year2 - The year (from 1-6) of the second measurement of SVL.

- year3 - The year (from 1-6) of the third measurement of SVL.

- year4 - The year (from 1-6) of the fourth measurement of SVL.

- year4 - The year (from 1-6) of the fifth measurement of SVL.

- t0 - A blank field for spacing growth intervals by year. All entries are zero.

- t1 - The interval between the first and second SVL measurements, in days.

- t2 - The interval between the second and third SVL measurements, in days.

- t3 - The interval between the third and fourth SVL measurements, in days.

- t4 - The interval between the fourth and fifth SVL measurements, in days.

- `all_rice_array.R` - A `.R` file containing an array with the proportion of the area undergoing active rice cultivation in each year for each site. Dimensions are sites x years x buffer distance. Buffer distances range from 0.1 km to 10 km in increments of 0.1 km.

- `all_buffer_area_array.R` - A `.R` file containing an array with the area contained within each buffer distance for each site. Dimensions are sites x years x buffer distance. Buffer distances range from 0.1 km to 10 km in increments of 0.1 km.

- `SPEI06_by_site.R` - A `.R` file containing a data frame with the 6-month standardized precipitation evapotranspiration index (SPEI06) for each site in August of each year from 2018 to 2023. This file includes:

  - site_code - A letter representing the site at which that snake was captured.

  - site_num - A numeric index for the site at which a snake was captured.

  - SPEI06.aug.2018 - SPEI06 values for August 2018 for each site.

  - SPEI06.aug.2019 - SPEI06 values for August 2019 for each site.

  - SPEI06.aug.2020 - SPEI06 values for August 2020 for each site.

  - SPEI06.aug.2021 - SPEI06 values for August 2021 for each site.

  - SPEI06.aug.2022 - SPEI06 values for August 2022 for each site.

  - SPEI06.aug.2023 - SPEI06 values for August 2023 for each site.

Required software

- R version 4.4.0 or later (R Core Team 2024) available at https://www.r-project.org/

Required R packages

- `coda` R package version 0.19-4.1 (Plummer et al. 2006)

- `dagitty` R package version 0.3-4 (Textor et al. 2016)

- `dplyr` R package version 1.1.4 (Wickham et al. 2019)

- `runjags` R package version 2.2.2-4 or later (Denwood 2016)

References

Chandler, R., and J. Hepinstall-Cymerman. 2016. Estimating the spatial scales of landscape effects on abundance. Landscape Ecology 31:1383–1394.

Denwood, M. J. 2016. runjags: An R package providing interface utilities, model templates, parallel computing methods and additional distributions for MCMC models in JAGS. Journal of Statistical Software 71:1–25.

Miguet, P., L. Fahrig, and C. Lavigne. 2017. How to quantify a distance-dependent landscape effect on a biological response. Methods in Ecology and Evolution 8:1717–1724.

Plummer, M., N. Best, K. Cowles, and K. Vines. 2006. CODA: convergence diagnosis and output analysis for MCMC. R News 6:7–11.

R Core Team. 2024. R: a language and environment for statistical computing. R Foundation for Statistical Computing, Vienna, Austria.

Riecke, T. V., A. G. Leach, D. Gibson, and J. S. Sedinger. 2018. Parameterizing the robust design in the BUGS language: Lifetime carry-over effects of environmental conditions during growth on a long-lived bird. Methods in Ecology and Evolution 9:2294–2305.

Rose, J. P., and B. J. Halstead. 2025. Code to estimate effects of rice growing on growth and survival of giant gartersnakes, *Thamnophis gigas*. U.S. Geological Survey Software Release. https://doi.org/10.5066/P145MXF4.

Rose, J. P., A. M. Nguyen, A. C. Jordan, D. A. Macias, E. J. Schoenig, G. R. Napolitano, R. Kim, J. S. M. Ersan, A. M. Fulton, and B. J. Halstead. 2024. Growth and capture mark recapture data from giant gartersnakes (*Thamnophis gigas*) in rice irrigation canals 2018 to 2023. https://doi.org/10.5066/P1JHAWVT.

Textor, J., B. van der Zander, M. S. Gilthorpe, M. Liśkiewicz, and G. T. Ellison. 2016. Robust causal inference using directed acyclic graphs: The R package "dagitty." International Journal of Epidemiology 45:1887–1894.

Wickham, H., R. François, L. Henry, and K. Müller. 2019. dplyr: A Grammar of Data Manipulation.

Table S1. Number of traplines at which giant gartersnakes (*Thamnophis gigas*) were sampled by site and year, and the maximum number of traplines sampled in any year (Max Traplines).

Site	Max Traplines	2018	2019	2020	2021	2022	2023
A	3	3	3	3	3	1	3
B	2	2	2	2	2	2	2
C	3	3	3	3	3	3	3
D	1	1	1	1	1	1	1
E	3	3	3	3	3	3	3
F	2	2	2	2	2	2	2
G	1	1	1	1	1	1	1
H	1	1	1	1	1	1	1
I	3	3	3	3	3	2	3
J	4	4	4	4	4	4	4
K	1	1	1	1	1	1	1
L	2	2	2	2	2	2	2
M	1	1	1	1	1	1	1
N	1	1	1	1	1	1	1
O	1	1	1	1	1	1	1
P	2	2	2	2	2	2	2
Q	2	2	2	2	1	1	0
R	1	1	1	1	1	1	1
S	5	5	5	3	3	3	3

Table S2. Accuracy of parameter estimates when robust-design CMR model is fitted to simulated data with known true parameter values. Priors are uniform distributions used to generate random true parameter values when generating simulated data. Bias is the mean difference between true value and mean posterior estimate, divided by the true value. Coverage is the proportion of 95% credible intervals that overlap the true value. Unif(min,max) is a Uniform distribution with minimum and maximum values.

Parameter	Description	Prior	Bias	Coverage
p	Daily recapture probability	Unif(0.025, 0.045)	0.00125	0.89
ϕ	Annual apparent survival probability	Unif(0.40, 0.60)	0.00722	0.99
γ	Availability for capture	Unif(0.45, 0.65)	0.00365	0.92
θ_{rice}	Effect of rice on ϕ	Uniform(0.30, 0.70)	-0.00134	0.96
$\sigma_{rice,\phi}$	Scale of rice effect on ϕ	Uniform(1, 9)	0.21005	0.98

Table S3. Parameters in the growth and capture-mark-recapture (survival) models for giant gartersnakes (*Thamnophis gigas*) and their priors and name used in JAGS code (JAGS name) by Rose and Halstead (2025).

Model	Parameter	Description	Prior	JAGS name
Growth	$\varepsilon_{a,fem}$	Asymptotic SVL for females	N(900,200)	eps.a.f
	$\varepsilon_{a,male}$	Asymptotic SVL for males	N(700,200)	eps.a.m
	$\mu_{k,fem}$	Log growth coefficient for females	N(0,10)	mu.k.f
	$\mu_{k,male}$	Log growth coefficient for males	N(0,10)	mu.k.m
	β_{rice}	Effect of rice on *k*	N(0,10)	beta.k.rice
	$\sigma_{rice,k}$	Scale of rice landscape covariate	Unif(0.01,10)	sigma.k.rice
	$\beta_{drought}$	Effect of drought on *k*	N(0,10)	beta.k.drought
	$\sigma_{ind,k}$	SD of individual random effect on *k*	Exp(1)	sd.ind.k
	$\sigma_{t,k}$	SD of year random effect on *k*	Exp(1)	sd.k.t
	$\sigma_{s,k}$	SD of site random effect on *k*	Exp(1)	sd.site.k
	$\sigma_{s,a}$	SD of site random effect on a	Exp(0.1)	sd.site.a
	$\sigma_{ind,L}$	SD of individual variation in measured SVL	Exp(0.1)	sd.eps
Survival	p_{fem}	Daily recapture probability for females	Beta(1,1)	mean.p[1]
	p_{male}	Daily recapture probability for males	Beta(1,1)	mean.p[2]
	$\sigma_{p,t}$	SD of year random effect on *p*	Exp(1)	sigma.p.t
	$\sigma_{p,s}$	SD of site random effect on *p*	Exp(1)	sigma.p.s
	θ_{rice}	Effect of rice on φ	*N*(0,10)	theta.rice.surv
	$\sigma_{rice,\phi}$	Scale of rice landscape covariate on φ	Unif(0.01,10)	sigma.rice.surv
	$\theta_{drought}$	Effect of drought on φ	*N*(0,10)	theta.drought.surv
	ϕ_{fem}	Annual apparent survival for females	Beta(1,1)	mean.phi[1]
	ϕ_{male}	Annual apparent survival for males	Beta(1,1)	mean.phi[2]
	$\sigma_{\phi,t}$	SD of year random effect on φ	Exp(1)	sigma.phi.t
	$\sigma_{\phi,s}$	SD of site random effect on φ	Exp(1)	sigma.phi.s
	γ_{fem}	Availability for females	Beta(1,1)	mean.gamma[1]
	γ_{male}	Availability for males	Beta(1,1)	mean.gamma[2]
	$\sigma_{\gamma,t}$	SD of year random effect on γ	Exp(1)	sigma.gamma.t

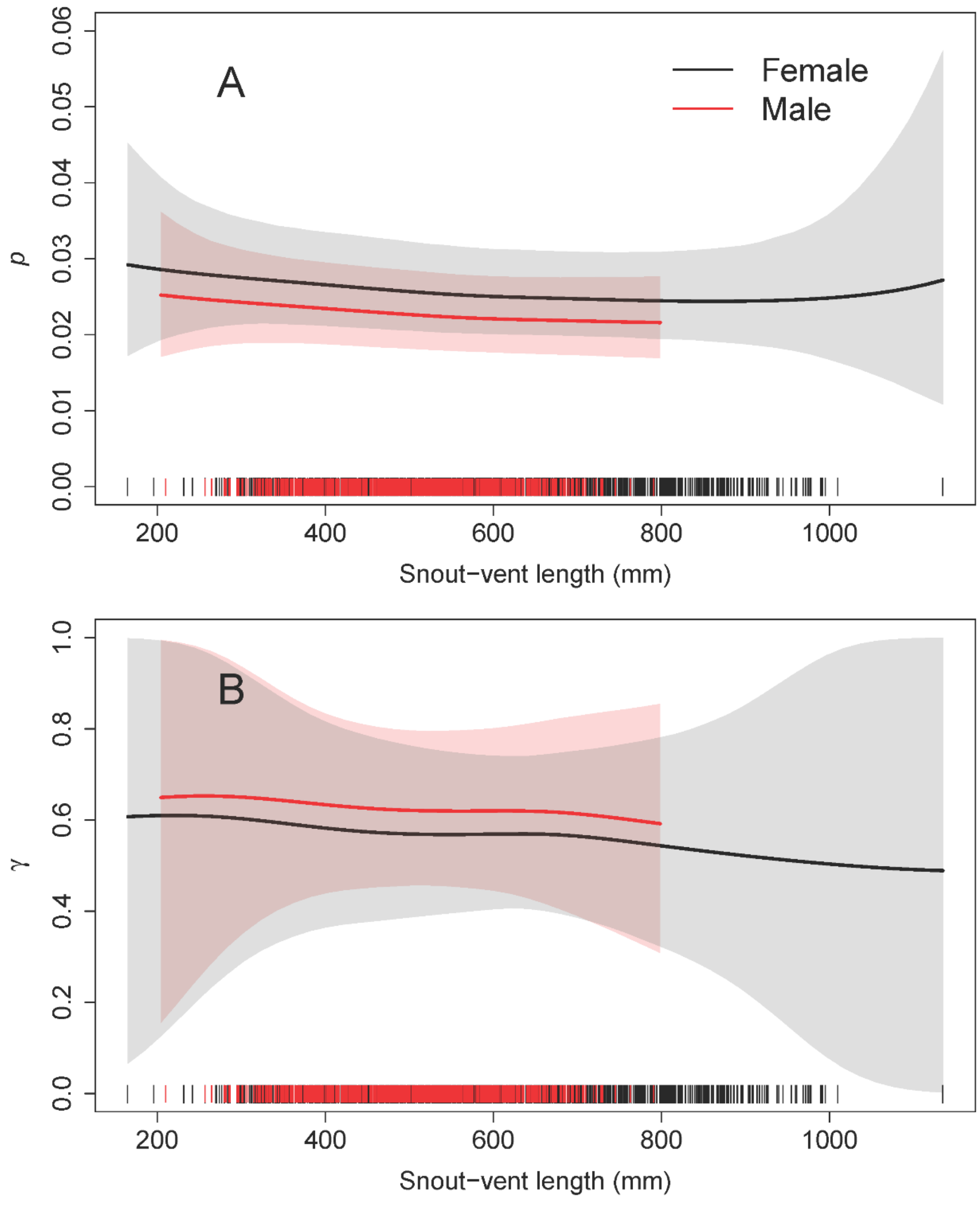

Figure S1. Relationships between giant gartersnake (*Thamnophis gigas*) size (snout-vent length, SVL in mm) and A) daily recapture probability (*p*) and B) availability for capture (γ) for females (black line, gray shading) and males (red line, red shading). Lines represent posterior mean predicted relationships, shaded regions represent equal-tailed 95% credible intervals. Tick marks above x-axis represent measured SVL values for females (black) and males (red). Predictions were limited to observed SVL values for each sex.

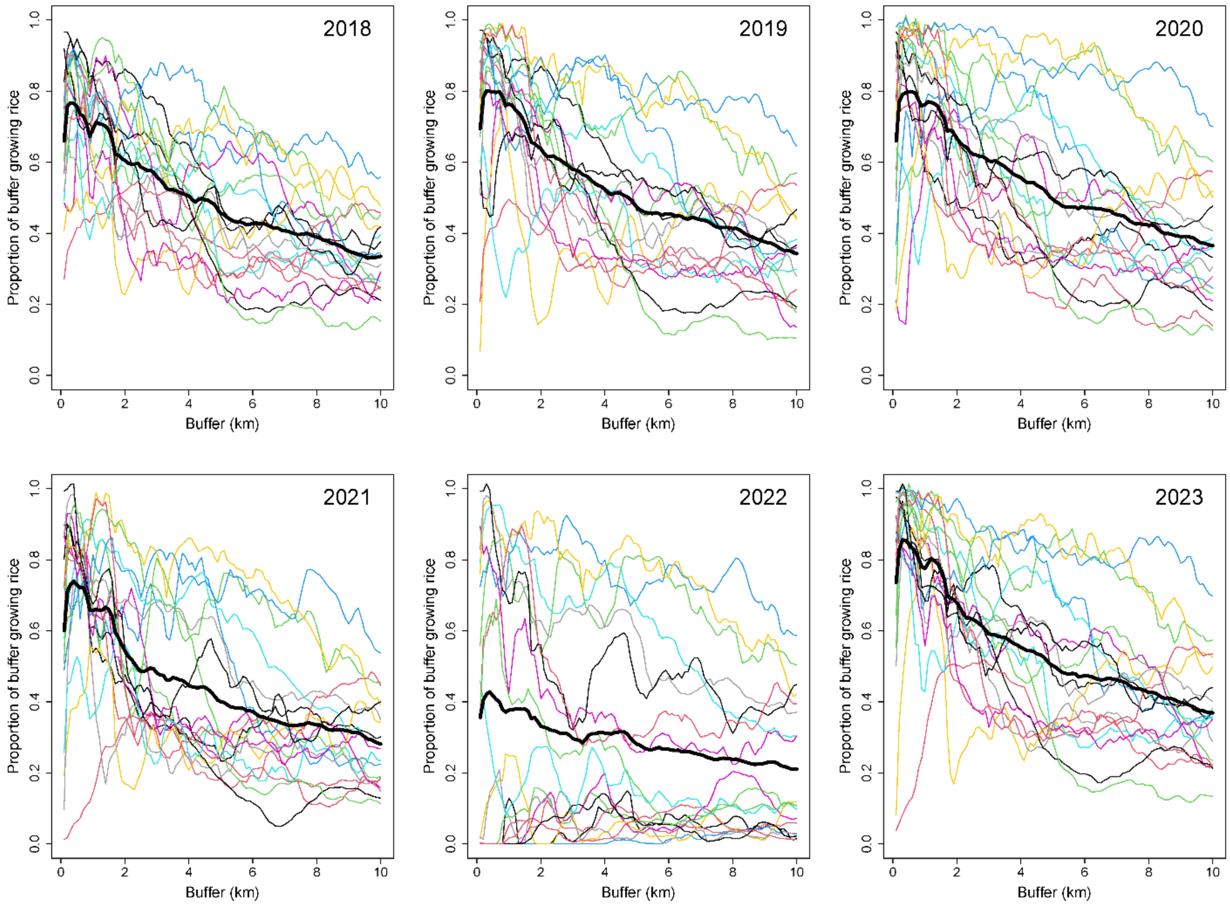

Figure S2. Proportion of the enclosed area in which rice was grown (y-axis) within buffers of varying distance (x-axis) at 19 sites in the Sacramento Valley, California from 2018 to 2023. Thin lines are proportion of rice grown at individual sites, the thick black line represents the average over all sites.

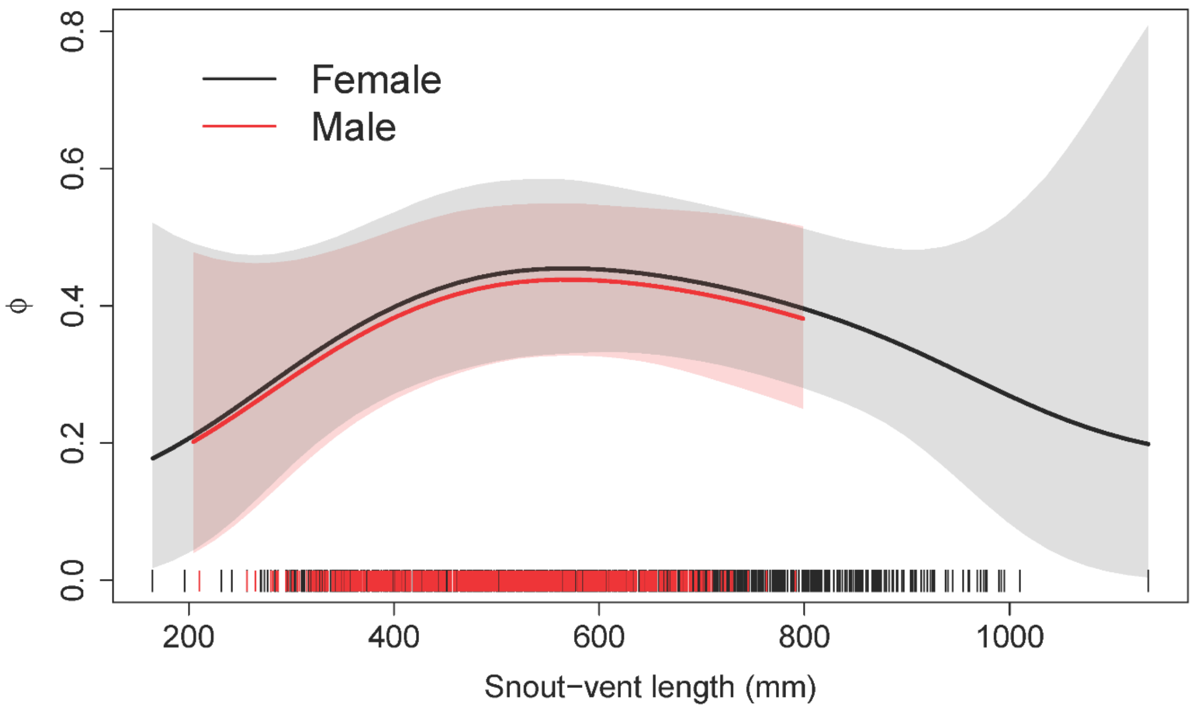

Figure S3. Relationships between giant gartersnake (*Thamnophis gigas*) size (snout-vent length, SVL in mm) and apparent survival (ϕ) for females (black line, gray shading) and males (red line, red shading). Lines represent posterior mean predicted relationships, shaded regions represent equal-tailed 95% credible intervals. Tick marks above x-axis represent measured SVL values for females (black) and males (red). Predictions were limited to observed SVL values for each sex.

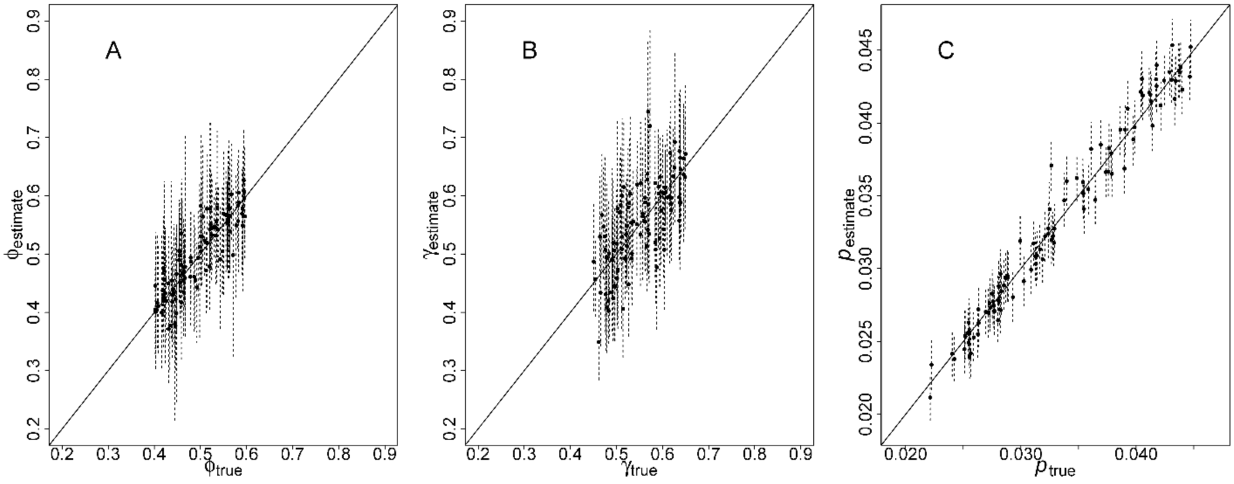

Figure S4. Accuracy of parameter estimates from the robust-design CMR model (y-axis) compared to true parameter values used to generate simulated data (x-axis). The solid diagonal line is a 1:1 line. Points are posterior mean values for parameter estimates on the y-axis, and dashed lines are 95% equal-tailed credible intervals. Panel A) apparent survival (ϕ), B) availability for capture (γ), C) daily recapture probability (*p*). Figure created by adapting R code from Riecke et al., (2018).

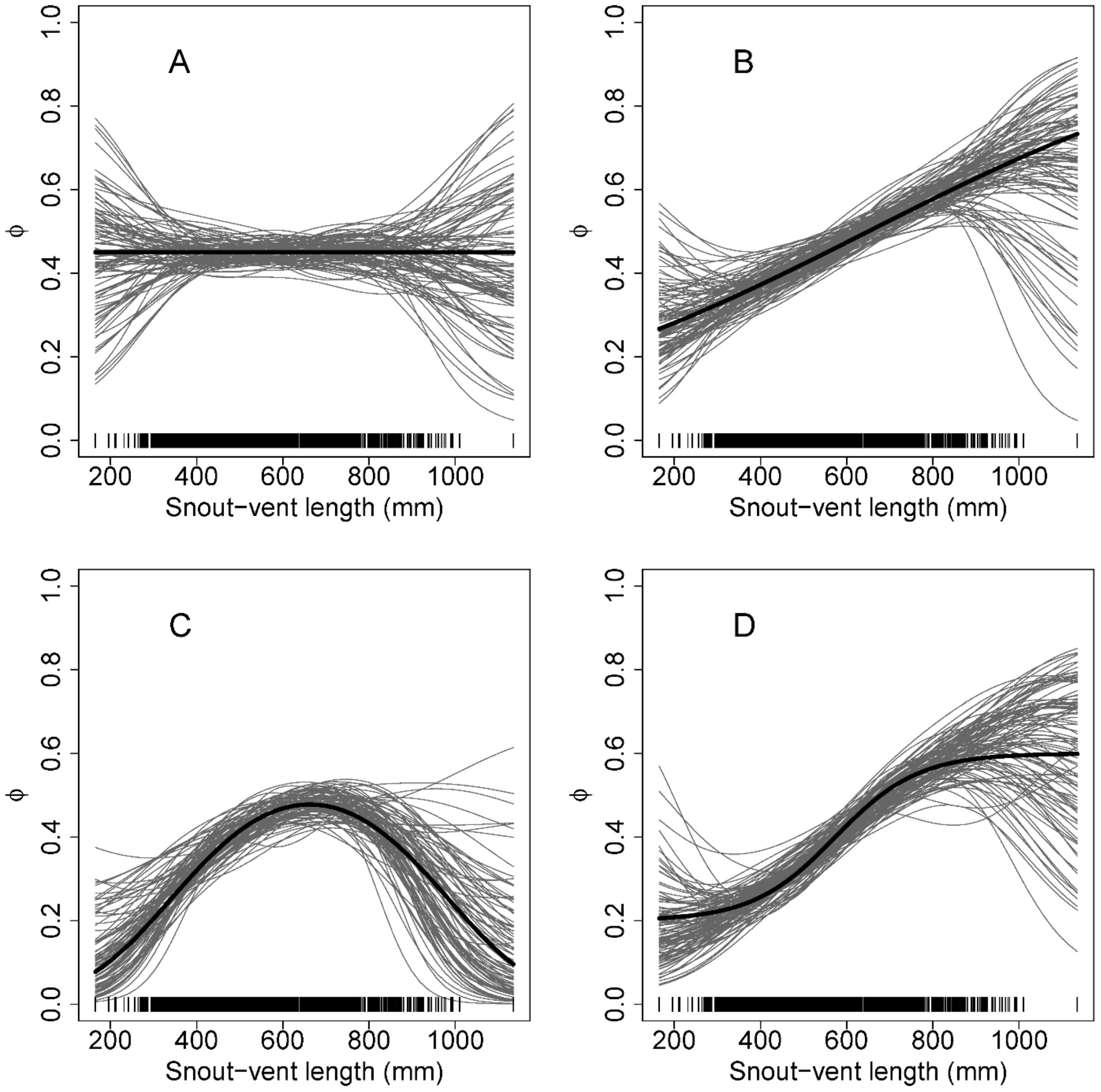

Figure S5. Simulated relationships between snake snout-vent length (SVL; x-axis) and apparent survival (ϕ; y-axis). True relationships used to simulate CMR data are represented by thick black lines, mean estimated relationships from the robust-design CMR model are thin gray lines. For each function, 100 simulations were run. Panel A) Size-independent survival (no relationship); B) Positive linear effect of SVL on survival; C) Quadratic effect of SVL on survival; D) Asymptotic positive effect of SVL on survival. All parameters, (p, ϕ, γ, β_{SVL}) were fixed to the same values for each simulated dataset for a given function. Tick marks above x-axis represent measured first SVLs for real giant gartersnakes, which were sampled (with replacement) to initialize SVL for simulated snakes.

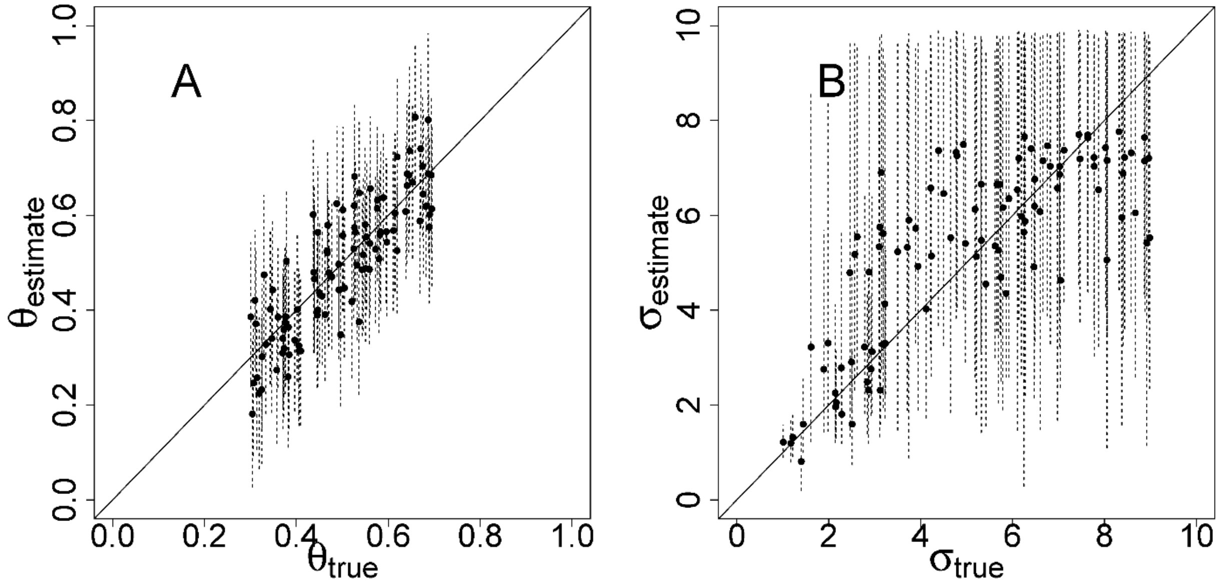

Figure S6. Accuracy of parameter estimates for rice effects on survival from the robust-design CMR model (y-axis) compared to true parameter values used to generate simulated data (x-axis). The solid diagonal line is a 1:1 line. Points are posterior mean values for parameter estimates on the y-axis, and dashed lines are 95% equal-tailed credible intervals. Panel A) The slope of the effect of rice on survival (β_{rice}), B) availability for capture (σ_{rice}). Figure created by adapting R code from Riecke et al., (2018).
